# Dried rhododendron flower ingestion presenting with bradycardia and hypotension: a case report

**DOI:** 10.1186/s13256-022-03413-8

**Published:** 2022-05-13

**Authors:** Sushil Baral, Binaya Kumar Baral, Pranit Sharma, Surendra Lal Shrestha

**Affiliations:** 1 Department of Internal Medicine, Bir hospital, National Academy of Medical Sciences (NAMS), Kathmandu, Nepal; 2 Department of Biochemistry, Nepal Medical College, Jorpati, Kathmandu, Nepal; 3 Vinayak Hospital and Maternity Home Pvt. Ltd, Kathmandu, Nepal

**Keywords:** Bradycardia, Hypotension, Rhododendron

## Abstract

**Background:**

Rhododendron toxicity can be a life-threatening situation when manifested; it results in bradycardia and hypotension. Treatment remains challenging when it is complicated with refractory hypotension involving the multiorgan system if not treated early.

**Case presentation:**

A 33-year-old Magar male presented with history of ingestion of two handfuls of white rhododendron flower. He had ingested the flowers believing that it would help relieve the pain and remove the materials stuck in his food pipe. Symptoms presented included muscular discomfort, dizziness, nausea, palpitation, tingling sensation around the face and lips, difficulty breathing, chest tightness, and difficulty swallowing within 4–5 hours after the ingestion of the dried flower. High-flow oxygen, intravenous fluids, atropine, and other supportive measures were used during the emergency, followed later by transfer to the intensive care unit for further observation.

**Conclusion:**

The patient was discharged with complete recovery after 2 days of hospital stay. Intentional or accidental ingestion of toxic plants can be severe or even life-threatening. Thus, clinicians should be familiar with local toxic plants with grayanotoxin action.

## Background

There are more than 33 species of rhododendron in Nepal, with dozens of varieties of all sizes and colors. The rhododendron arboreum (*Lali Gurans* in Nepali) is the most famous among the different species and is a national flower. Grayanotoxins from certain rhododendron species result in acute intoxication that is manifested as hypotension, bradycardia, nausea, vomiting, and dizziness. This intoxication is well known in the Black Sea regions of Turkey since wild honey produced by bees in this area is often derived from *Rhododendron ponticum* or *Rhododendron luteum*, which contain grayanotoxins [1, 2].

## Case presentation

### Patient information

We describe the case of a 33-year-old Magar male with complaints of muscular discomfort, dizziness, nausea, and tingling sensation around the face and lips. He also felt difficulty breathing and chest tightness and difficulty swallowing, after ingesting two handfuls of dried white rhododendron flower (approximately 40–50 g). As stated by the patient and his party, he had orally ingested the white rhododendron flower. This is a trend practiced in his village for removing pieces of bone or other materials stuck in the food pipe. The patient presented approximately 4 hours after consumption with the symptoms mentioned above.

### Clinical findings

On examination, he was conscious, alert, and well oriented. His heart rate was 40–48 beats/minute and regular with blood oxygen saturation (SpO_2_) of 94% in room air and blood pressure (BP) of 70/40 mmHg. Clinical examination revealed no murmur or gallop, and chest was clear with no added sounds. He also denied consumption of alcohol or any other drugs. Similarly, there was no history of diabetes, hypertension, thyroid, or any illness or cardiac conditions. Also, there was no sign of head trauma or focal neurological symptoms.

### Timeline

He was immediately observed in the emergency department, received medical attention, and transferred to the intensive care unit (ICU) for further observation.

### Diagnostic assessment

**Fig. 1:**
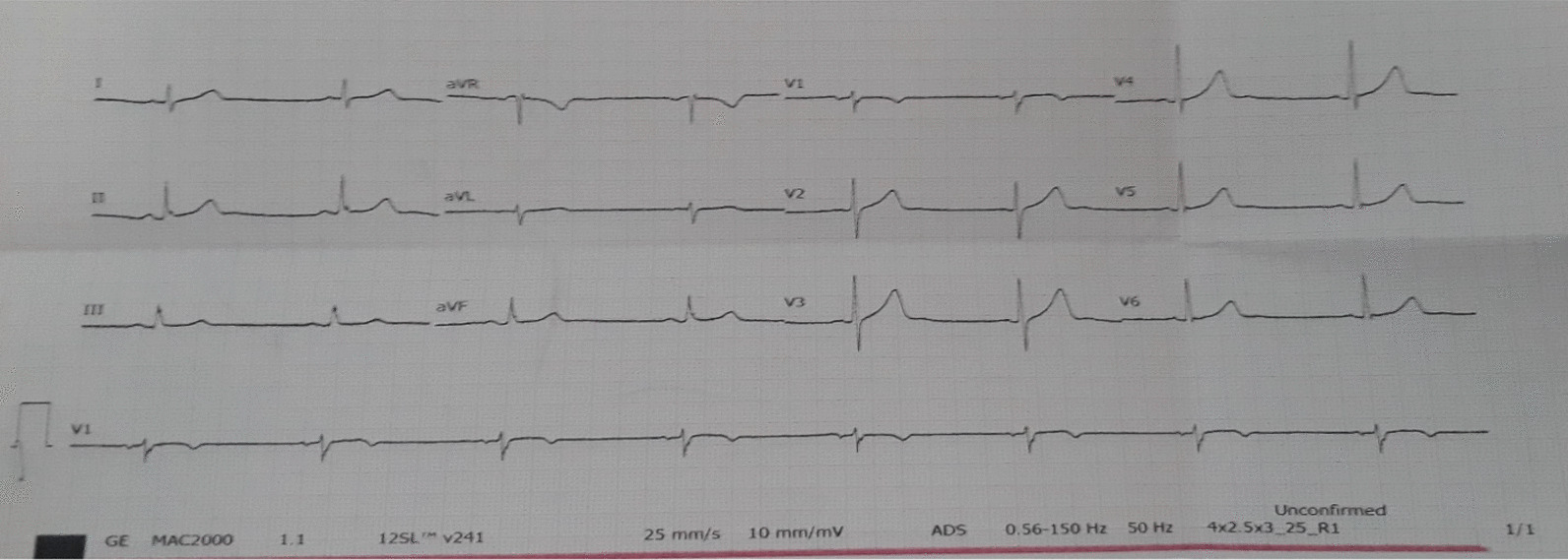
Electrocardiogram taken at time of admission shows sinus bradycardia with a heart rate of 47 beats/minute

His initial complete blood cell count (CBC), renal function tests, electrolytes, and thyroid function test (TFT) were unremarkable. An electrocardiogram (ECG) showed sinus bradycardia with a heart rate (HR) of 47 beats/minute (Fig. 1).

**Fig. 2:**
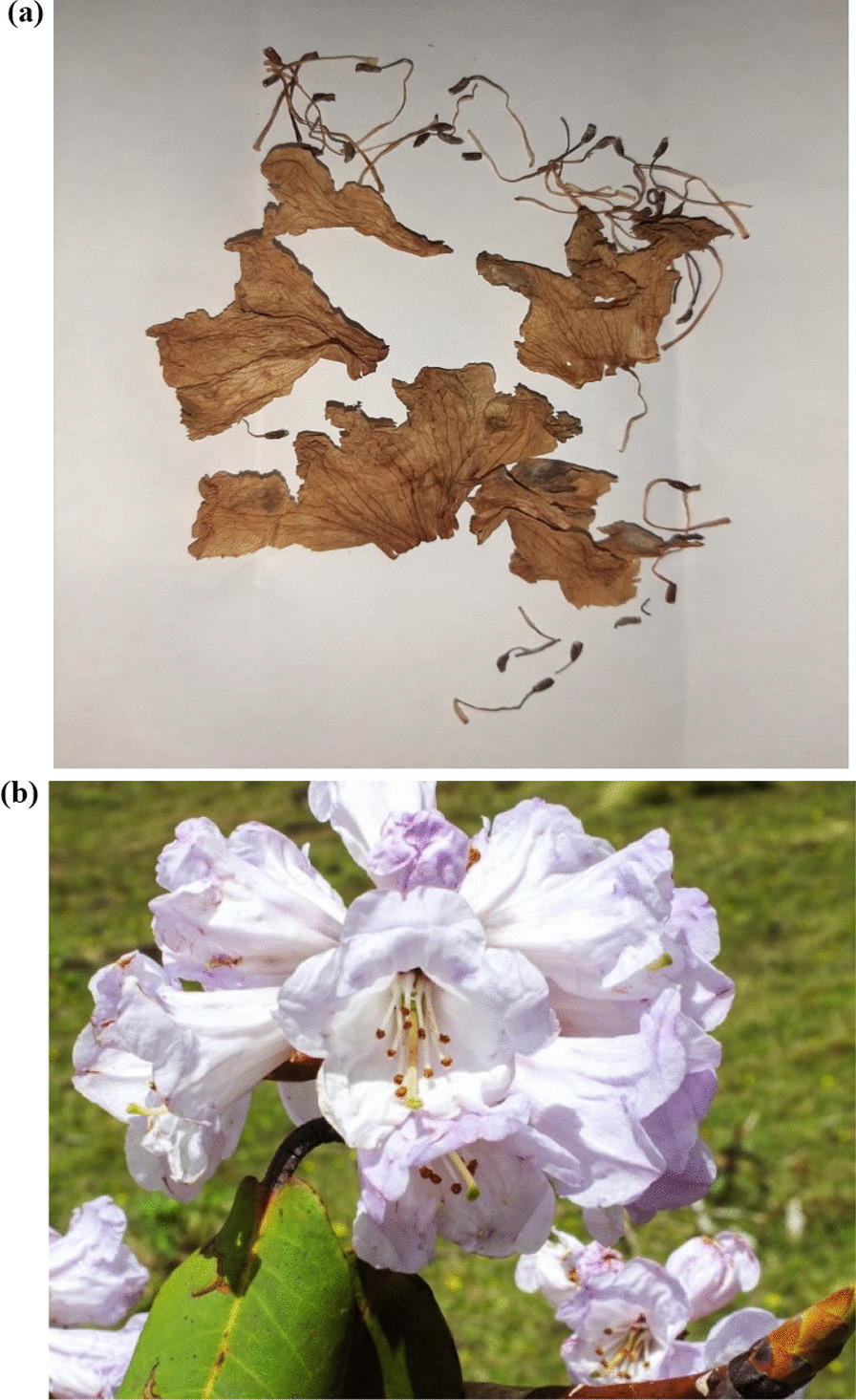
**a** Rhododendron (remnant of flower that the patient had ingested). **b** Color picture of rhododendron flower

Cardiac markers were negative. Chest X-ray and ultrasound (USG) of the abdomen revealed no significant abnormality on admission. An echocardiogram revealed normal left ventricle (LV) and right ventricle (RV) function. His family had brought the remnant of the flowers he had ingested (Fig. 2a).

According to the clinical course, acute intoxication by some species of rhododendron was suspected.

### Treatment or intervention

**Fig. 3:**
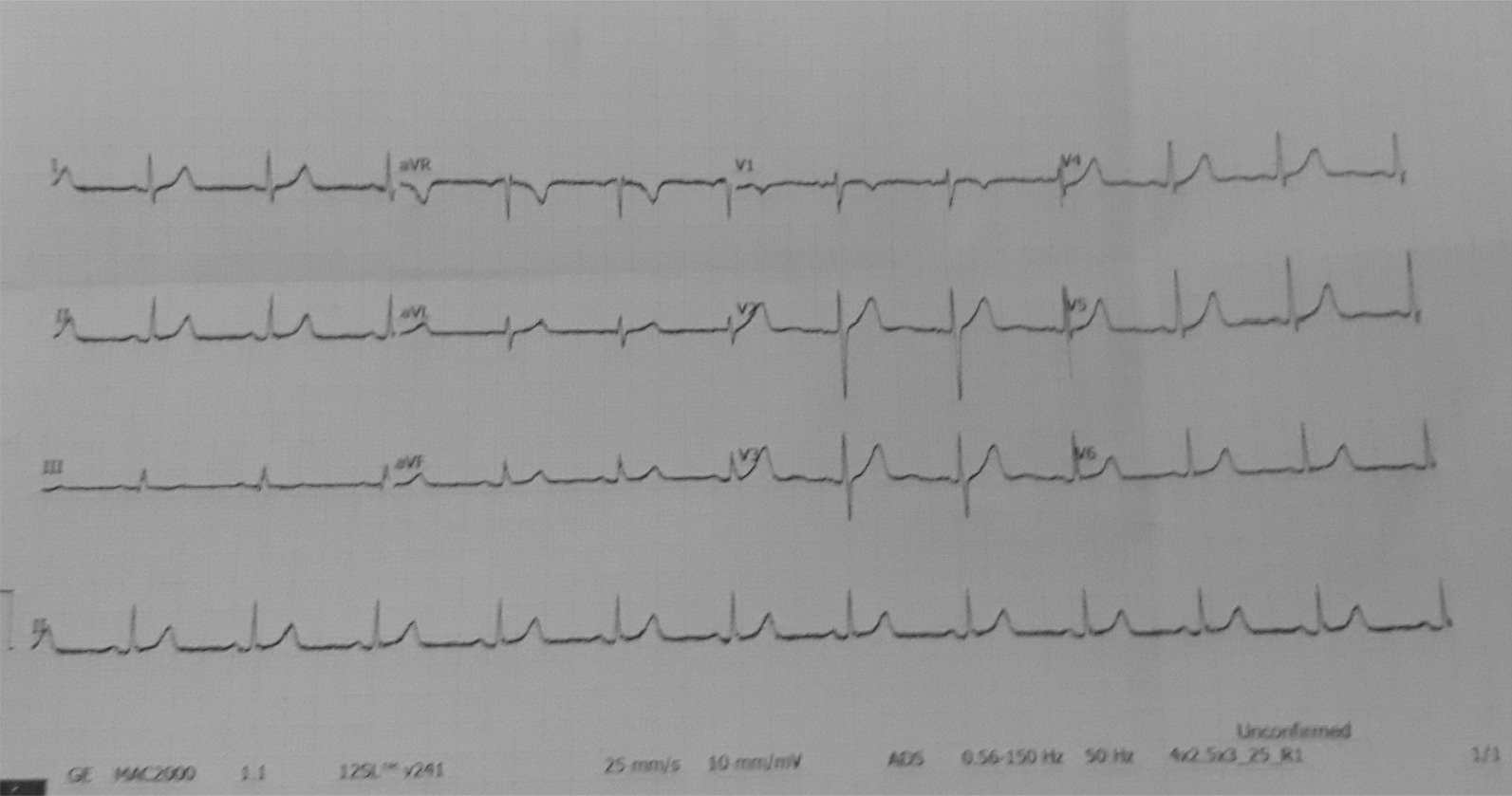
Electrocardiogram taken 24 hours later shows normal sinus rhythm with heart rate of 86 beats/minute

He has continued on normal intravenous saline at 50 mL/hour, and at the same time 2 mg atropine was given when he was in ICU. After 24 hours of hospital stay, he showed gradual improvement in his clinical condition with BP 110/70 mmHg and heart rate of 76 beats/minute. An electrocardiogram carried out 24 hours after admission showed normal sinus rhythm with heart rate of 86 beats/minute (Fig. 3). After 48 hours of admission, he was discharged in good health without any clinical symptoms.

## Discussion

Grayanotoxin is found in rhododendrons and other plants of the family Ericaceae. It is also known that ingestion of certain members of Ericaceae, that is, rhododendron species, causes acute intoxication by toxic substances called grayanotoxins. This intoxication is well known in the Black Sea regions of Turkey as “mad honey poisoning” since beekeeping and honey production have been historically practiced in this area. Consumption of wild honey produced from grayanotoxin-containing plants such as *Rhododendron ponticum* leads to this acute intoxication [1, 2]. There are various forms of grayanotoxin. The specific grayanotoxin varies with the plant species. Grayanotoxin I is the principal toxic isomer [3].

The underlying mechanism is binding to sodium channels in the cell membrane, preventing their inactivation. Excitable tissues, including nerve and muscle cells, are thus maintained in a state of depolarization, during which entry of calcium into the cells may be facilitated. This action is similar to that of the aconitum alkaloids, another group of plant toxins [4, 5].

Grayanotoxin has adverse effects on the cardiovascular system, including systemic hypotension, bradycardia, and atrioventricular block. A previous case series on grayanotoxin poisoning reported bradycardia and hypotension in 15 of 19 patients. In two patients, bradycardia was severe, and four patients had complete atrioventricular block [6]. Other symptoms such as nausea, vomiting, sweating, salivation, dizziness, weakness, blurred vision, convulsions, and loss of consciousness are also seen. Our patient presented with bradycardia, hypotension, and excessive salivation along with tingling and numbness around the mouth and lips, with chest tightness and difficulty in swallowing, which are all consistent with grayanotoxin poisoning [1, 2].

Rhododendron is widely found in the mountains of Nepal. Various studies show that common symptoms observed after ingestion of grayanotoxin-containing material leads to hypotension and bradyarrhythmia [2]. Bradyarrhythmia may be sinus bradycardia, complete atrioventricular block, or atrial fibrillation with slow ventricular response [2, 6].

The treatment for this intoxication is supportive. Intravenous atropine and norsmal saline infusion are typically sufficient, with the aim of full recovery in 24 hours. In the present case, in addition to severe nausea and vomiting, hypotension and bradycardia were evident. Gunduz *et al.* reported that hypotension and bradycardia were observed with grayanotoxin poisoning [1].

We suggest that, although this is a rare condition, acute intoxication by grayanotoxin-containing plants should be considered if patients present with unexplained hypotension and bradycardia after ingestion of various botanical plants available in our country.

Clinicians should be aware that some species of rhododendron contain grayanotoxin, causing acute intoxication with clinical manifestation. Among patients presenting with nausea, vomiting, and abdominal pain with unexplained hypotension and/or bradycardia, a detailed dietary history is recommended for differential diagnosis of grayanotoxin poisoning [3]. Confirmation of plant poisoning is difficult in country with less resources such as Nepal. Thus, morphological identification is helpful if the plant and flowers are available. However, not all rhododendron species are toxic, and a specific chemical analysis is needed to identify the toxin, which is essential for the confirmation of human poisonings. Laboratory studies help us confirm plant toxins and exclude other causes of poisoning, although we were not able to confirm the toxin by chemical analysis in this case.

## Conclusions

After 48 hours of admission, the patient was discharged in good health without any clinical symptoms. Similarly, he also said that he would never take any local herbal leaves or flowers without consulting the doctor next time. Thus, the community should be discouraged from using unknown botanical plants and flowers as is often practiced in our country because of various myths. Early identification and supportive therapy are the keystones of successful treatment, and clinicians should be aware of locally available poisonous plants and their toxicity.

## Data Availability

The relevant laboratory investigations are available in the medical record of the hospital and can be assessed if required.

## Abbreviations

BP: Blood pressure
CBC: Complete blood cell count
ECG: Electrocardiogram
HR: Heart rate
ICU: Intensive care unit
IV: Intravenous
LV: Left ventricle
RV: Right ventricle
SpO_2_: Oxygen saturation
TFT: Thyroid function test
USG: Ultrasound

## References

[1] Gunduz A, Turedi S, Russell RM, Ayaz FA. Clinical review of grayanotoxin/mad honey poisoning past and present. Clin Toxicol. 2008;46:437–42. 10.1080/15563650701666306 18568799

[2] Koda R, Honma M, Suzuki K, Kasai A, Takeda T, Narita I, Yoshida K. Hypotension and bradycardia caused by the inadvertent ingestion of *Rhododendron japonicum*. Intern Med. 2016;55:839–42. 10.2169/internalmedicine.55.6144 27041175

[3] Scott PM, Coldwell BB, Wiberg GS. Grayanotoxins. Occurrence and analysis in honey and a comparison of toxicities in mice. Food Cosmet Toxicol. 1971;9:179–84. 10.1016/0015-6264(71)90303-8 5559997

[4] Kimura T, Kinoshita E, Yamaoka K, Yuki T, Yakehiro M, Seyama I. On-site of action of grayanotoxin in domain 4 segment 6 of rat skeletal muscle sodium channel. FEBS Lett. 2000;465:18–22. 10.1016/s0014-5793(99)01715-9 10620699

[5] Poon WT, Ho CH, Yip KL, Lai CK, Cheung KL, Sung RY, Chan AY, Mak TW. Grayanotoxin poisoning from *Rhododendron simsii* in an infant. Hong Kong Med J. 2008;14:405–7. 18840915

[6] Oguzturk H, Ciftci O, Turtay MG, Yumrutepe S. Complete atrioventricular block caused by mad honey intoxication. Eur Rev Med Pharmacol Sci. 2012;16:1748–50. 23161051

